# Evaluation of Respiratory Intensive Care Experiences and Relationships with Nutritional Status Among Patients Admitted to the Intensive Care Unit

**DOI:** 10.3390/medicina61020356

**Published:** 2025-02-19

**Authors:** Murat Yildiz, Deniz Celik, Tarkan Ozdemir, Kerem Ensarioglu, Melek Cakir, Tugce Dondu Savur, Oral Mentes, Mustafa Ozgur Cirik

**Affiliations:** 1 Department of Pulmonary Medicine, Atatürk Sanatorium Research Hospital, Faculty of Medicine, Health Sciences University, Ankara 06290, Turkey; tabiptarkan@hotmail.com (T.O.); kerem.ensarioglu@gmail.com (K.E.); cakir.melek2396@gmail.com (M.C.); tugcesavasir@gmail.com (T.D.S.); dr.ozgurr@hotmail.com (M.O.C.); 2 Department of Pulmonary Medicine, Faculty of Medicine, Alaaddin Keykubat University, Antalya 07425, Turkey; drdenizcelik@hotmail.com; 3 Intensive Care Unit, Gulhane Training and Research Hospital, Ankara 06010, Turkey; omentes@live.com

**Keywords:** respiratory intensive care unit, nutritional support, patient experience, ICU outcomes, psychosocial factors in intensive care

## Abstract

*Background and Objectives*: This study aimed to evaluate patients’ experiences in the intensive care unit (ICU) setting and investigate whether there was a correlation between these experiences and their nutritional status. *Materials and Methods*: This study included patients admitted to the respiratory ICU between 1 January 2023 and 31 December 2023. Only patients aged 18 years or older were eligible for inclusion. Written and verbal consent was obtained from all participants, while those unable to provide nonverbal communication were excluded from the study. The Intensive Care Experience Questionnaire (ICEQ), developed by Rattray et al. in 2004, was utilized to assess the overall experiences of ICU patients. *Results*: The ICEQ results were analyzed across four categories: awareness of surroundings, recall of experiences, frightening experiences, and satisfaction with care. A total score was generated by summing the scores of these four categories. While the initial parameters were analyzed as ordinal data, the results for the four subcategories and the total score followed a parametric distribution and were thus analyzed accordingly. *Conclusions*: These findings reinforce the hypothesis that nutritional support requirements play a critical role in shaping patients’ experiences in the ICU, regardless of their preadmission status. Adequate nutritional support was shown to have a positive effect on ICU experience.

## 1. Introduction

Intensive care units (ICUs) are specialized hospital wards dedicated to providing vital functional support for patients in critical condition. These units rely on highly skilled, multidisciplinary medical teams that employ a wide range of medical interventions and advanced devices. The primary goal of ICU admission is not only to sustain life but also to provide physiological and psychological support, ensuring that patients can recover and leave the ICU with positive experiences [1]. However, several factors may contribute to negative experiences in the ICU, including an unfamiliar environment, disrupted sleep patterns, immobilization, restrictions on visits from relatives, insufficient information about treatment plans, and frequent invasive procedures [2,3].

Critically ill patients often require additional life support interventions, such as respiratory, cardiovascular, or renal support. Despite the critical nature of their condition, ICU patients remain susceptible to emotional and psychological challenges. Many reported experiencing vivid dreams, hallucinations, and significant emotional fluctuations during their stay [4].

Recent studies have highlighted the profound impact of nutritional status on the outcomes of critically ill patients, influencing both physiological recovery and psychological well-being. Malnutrition, which is often prevalent in ICU patients due to metabolic stress, inadequate intake, and the catabolic nature of critical illness, has been associated with prolonged hospital stays, increased risk of infections, impaired wound healing, and increased mortality rates. Conversely, appropriate nutritional support has been shown to enhance immune function, preserve muscle mass, and potentially improve overall patient experiences during ICU admission [5,6]. Given these associations, understanding the interplay between nutritional status and ICU experiences is essential for optimizing patient-centered care and improving long-term recovery outcomes. This study aimed to assess the overall experiences of patients in an ICU setting and to explore whether a correlation exists between patients’ nutritional status and their ICU experiences.

## 2. Materials and Methods

### 2.1. Informed Consent

Before initiating our study, we ensured that the informed consent forms, which we routinely obtained from patients and/or their legal guardians in our intensive care units, were fully completed and approved. These forms grant permission for the use of patients’ clinical and radiological data in scientific research. The study was conducted in accordance with the Helsinki Declaration and was approved by the local ethics committee (dated 14 December 2022, decision number: 2012-KEAK 15/2607). Written and verbal consent was obtained from all participants, while those unable to provide verbal communication were excluded from the study.

### 2.2. Inclusion and Exclusion Criteria

Our study was designed and conducted in a secondary-level respiratory ICU, where the diagnoses primarily consist of decompensated respiratory acidosis and hypoxemia due to various lung diseases. The cognitive functions of these patients are generally better compared to those followed in tertiary-level general ICUs.

The nutritional protocol in our ICU is as follows:

- Patients who have intact swallowing function and are conscious are fed orally under the supervision of a clinical dietician, with protein and calorie calculations tailored to their needs.
- Patients with intact swallowing function who are unable to consume more than 50% of the calculated caloric requirement receive formulated oral nutritional solutions.
- Patients with impaired swallowing function or aspiration risk are fed via a nasogastric tube with formulated enteral solutions.
- Patients for whom enteral nutrition is contraindicated and whose condition is expected to last for more than one week are provided total parenteral nutrition (TPN) supplemented with trace elements and vitamins.

#### 2.2.1. Inclusion Criteria

- Patients were admitted to the pulmonary medicine intensive care unit (ICU) between 1 January 2023 and 31 December 2023.
- ICU stay exceeding 24 h.
- Patients aged 18 years or older.

#### 2.2.2. Exclusion Criteria

- Patients who were unable to communicate verbally and lacked cooperation and orientation due to any neurological disease or deficit.
- Patients with incomplete medical records.
- Patients were discharged or transferred within 24 h of ICU admission.

### 2.3. Data Collection

The data collection process was conducted via patient information and a follow-up form consisting of two components:

#### 2.3.1. General Characteristics and Comorbidities

Demographic and clinical data, including admission protocol number, sex, date of birth, marital status, primary diagnosis, comorbidities, admission and discharge dates, total hospitalization duration, source of admission, noninvasive mechanical ventilation (NIV) requirements, body mass index (BMI), and educational status, were collected.

#### 2.3.2. Laboratory Analyses

Routine blood parameters, such as hemoglobin, hematocrit, creatinine, urea, total protein, albumin, potassium, sodium, calcium, and magnesium, were measured. Nutritional evaluations were undertaken, including nutritional support requirements, type of nutritional support (parenteral or enteral), and nutritional scoring systems.

### 2.4. Intensive Care Experience Questionnaire (ICEQ)

To assess patients’ experiences in the ICU, the intensive care experience questionnaire (ICEQ), originally developed by Rattray et al. in 2004, was employed [7]. The ICEQ was translated into Turkish by Demir et al., with a reported Cronbach’s alpha reliability score of 0.79 [8]. The questionnaire consists of 19 Likert-type items, with nine focusing on patients’ adaptation to the ICU environment and the remaining ten assessing emotional states. The adaptation items were rated on a scale from 1 (“Strongly Disagree”) to 5 (“Strongly Agree”), whereas the emotional state items were rated from 1 (“Never”) to 5 (“Always”). Reverse scoring was applied to items 7, 8, 9, 10, 15, and 17. The total ICEQ score ranges from 19 to 95, with lower scores indicating poorer awareness and clarity of mind in the ICU and reflecting an overall unfavorable experience [8]. Additionally, the ICEQ includes four subcategories: “awareness of surroundings” (6–30 points), “recall of experiences” (4–20 points), “frightening experiences” (5–25 points), and “satisfaction with care” (4–20 points).

### 2.5. Statistical Analyses

Data analysis was performed after the collection and finalization of patient information via Microsoft Excel. Descriptive statistics are presented as counts (n), percentages (%), standard deviations (SDs), and means or medians, as appropriate. The normality of the parametric distributions was assessed via Q–Q plots. A paired sample *t*-test was applied to compare the admission and discharge parameters. Pearson correlation was used to analyze relationships between two continuous variables with parametric distributions, whereas Spearman correlation was used for nonparametric data.

Linear regression analysis was conducted to identify independent factors among parameters with statistically significant *p* values. Model validity was confirmed via the Hosmer–Lemeshow test. Statistical significance was defined as a *p* value of less than 0.05. All the statistical analyses were performed via IBM SPSS Statistics, version 23.

## 3. Results

A total of 171 patients were included in the study. The majority of the patients were male (*n* = 113, 66.1%), with an average age of 68.25 years (±11.28) and an average body mass index (BMI) of 24.28 (±3.78). The dominant educational level among the patients was primary school (*n* = 125, 73.1%). Hypertension was the most common comorbidity (*n* = 75, 43.9%), followed by diabetes mellitus (*n* = 43, 25.1%) and congestive heart failure (*n* = 30, 17.5%).

The average duration of hospitalization was 10.3 days (±6.2), with noninvasive mechanical ventilation (NIV) support required for a significant proportion of the patients (*n* = 108, 63.2%). Nutritional support, either enteral or parenteral, was necessary for approximately 15% of the patients.

Most ICU admissions were from either emergency services (*n* = 77, 45%) or other intensive care units (*n* = 77, 45%). A detailed breakdown of patient characteristics and clinical parameters is provided in Table 1.

Hemoglobin levels slightly decreased between admission and discharge. The creatinine level remained at approximately 1 mg/dL, with corresponding blood urea nitrogen (BUN) levels ranging from 50.08 mg/dL to 48.12 mg/dL. Electrolyte levels, including those of sodium, chloride, and potassium, were within normal ranges, whereas magnesium levels were stable at approximately 2 mEq/L.

Albumin and total protein levels were also slightly reduced. However, no statistically significant differences were observed in any laboratory parameters between admission and discharge. A detailed summary of the laboratory evaluations is provided in Table 2.

The results of the ICEQ were analyzed across four categories, with each question categorized under its respective domain. The four categories were awareness of surroundings, recall of experiences, frightening experiences, and satisfaction with care, which consisted of five, four, six, and four criteria, respectively.

The scores from these categories were summed to calculate a total ICEQ score. While the initial parameters were evaluated as ordinal, the results of the four subcategories and the total score demonstrated a parametric distribution and were analyzed accordingly. A detailed breakdown of the results is provided in Table 3.

Pearson correlation analysis was conducted to evaluate relationships between parameters across the four subgroups and the total score of the questionnaire. While no parameter was significantly correlated with the total score, several parameters demonstrated correlations with the four subgroups.

Age was negatively correlated with awareness of surroundings, satisfaction with care, and recall of experiences but positively correlated with frightening experiences. Nutritional support exhibited a correlation pattern similar to that of age, with negative correlations in the first three subgroups and a positive correlation in the last subgroup.

In contrast, the albumin level was inversely correlated with nutritional support, whereas the total protein level was not significantly correlated with any of the four subgroups. A detailed summary of the correlations is presented in Table 4.

As the number of hospitalization days increased, a negative correlation was observed between awareness of surroundings and satisfaction with care, whereas a positive correlation became evident with frightening experiences. A history of noninvasive mechanical ventilation (NIMV) showed a single negative correlation, specifically with recall of experiences.

The presence of diabetes mellitus and congestive heart failure (CHF) was negatively correlated with satisfaction with care, with CHF also showing a positive correlation with frightening experiences. Among the laboratory parameters, BUN (blood urea nitrogen) demonstrated the strongest correlations, whereas the other electrolytes exhibited only isolated positive or negative correlations (Table 4a,b).

### 3.1. Linear Regression Analysis

Linear regression analyses were performed to evaluate each subgroup of the questionnaire independently. All models had statistically significant results and demonstrated acceptable Durbin–Watson values (between 1.7 and 2.0). The regression and residual degrees of freedom, F statistics, and *p* values for each subgroup were as follows:

- Awareness of surroundings: F(19,149) = 3.639, *p* = 0.001
- Recall of experiences: F(19,149) = 2.389, *p* = 0.001
- Frightening experiences: F(19,149) = 4.117, *p* = 0.001
- Satisfaction with care: F(19,149) = 1.837, *p* = 0.023

The models had R values of 0.563, 0.483, 0.587, and 0.436 and adjusted R^2^ values of 0.230, 0.136, 0.261, and 0.086 for awareness of surroundings, recall of experiences, frightening experiences, and satisfaction with care, respectively. The regression analysis revealed that the least robust model was for satisfaction with care, as indicated by its lower R and adjusted R^2^ values.

Across all the models, nutritional support was identified as an independent factor affecting subgroup outcomes. Nutritional support was positively associated with frightening experiences (*p* = 0.001) but negatively associated with awareness of surroundings (*p* = 0.001), recall of experiences (*p* = 0.000), and satisfaction with care (*p* = 0.042).

### 3.2. Additional Observations

- Gender: Female patients reported greater awareness of their surroundings than male patients did (*p* = 0.038).
- Age: Older individuals were more likely to report frightening experiences (*p* = 0.047).
- Magnesium: Among laboratory parameters, magnesium was the only independent factor. Lower magnesium levels were significantly correlated with reduced awareness of surrounding areas (*p* = 0.002).

The detailed results of the regression analysis and correlation evaluations are presented in Table 5 and Table 6.

## 4. Discussion

The laboratory results of the patients were similar at admission and discharge. When these results are considered alongside the effects on the questionnaire findings, this observation supports the idea that admission values may serve as a reliable basis for analyzing the influence of nutritional parameters rather than relying on discharge values. Consequently, predicting questionnaire outcomes and patient satisfaction with care may be feasible as early as ICU admission. However, as indicated by the regression analysis, the role of nutritional parameters (e.g., albumin and total protein) becomes less prominent than the actual presence of nutritional support. This is evident in the lack of correlation between total protein and questionnaire outcomes and the absence of a relationship between albumin levels and nutritional support, which remained a relevant factor regardless of the model or subgroup.

Hypoalbuminemia has been established as an independent risk factor for unfavorable clinical outcomes, as shown in a meta-analysis. Patients with hypoalbuminemia, especially those admitted to the ICU or general wards with a history of surgery or renal dysfunction, often experience adverse outcomes. These include increased mortality, morbidity, and prolonged intensive care unit (ICU) and hospital stays [9]. Furthermore, hypoalbuminemia has been shown to be associated with infection severity and sepsis development, particularly in critically ill patients, as systemic inflammation leads to increased capillary permeability and alterations in albumin metabolism [10]. Additionally, in patients with hypercapnic respiratory failure, hypoalbuminemia has been identified as a significant predictor of prolonged ICU stays, where lower albumin levels are associated with increased morbidity and susceptibility to infections [11]. The diverse etiologies of hypoalbuminemia suggest that it may be a compensatory mechanism that does not always require intervention. However, reductions in osmotic pressure, intravascular antioxidative reserve, and other protective effects justify the potential use of albumin supplementation to prevent worsening outcomes, even though hypoalbuminemia itself serves as a marker of pathological processes [9,12,13].

Cost-effective strategies aimed at reducing hospitalization and ICU durations are increasingly relevant as the number of patients requiring end-of-life care and managing comorbidities increases. Predictive models for estimating hospital length of stay have identified hypoalbuminemia, ICU requirements (excluding cardiovascular ICUs), advanced age, prior hospitalizations, pressure ulcers, and early mechanical ventilation as significant factors [14]. These findings highlight the potential role of variables such as mechanical ventilation, age, and comorbidities in ICU discharge evaluations beyond traditional predictors.

Chen et al. reported that patients with chronic lung diseases or hypertension had shorter ICU stays than other patients did, suggesting that these conditions may increase mortality to the extent that hospitalization durations are shortened [15]. Interestingly, our findings indicate that nutritional support is negatively correlated with awareness of surroundings and satisfaction with care. One possible explanation is that patients requiring nutritional support may have had more severe conditions, leading to lower awareness due to critical illness, sedation, or prolonged hospitalization. Additionally, these patients might have experienced greater physical discomfort or psychological distress, potentially influencing their perception of care negatively. This finding highlights the need for improved nutritional support strategies that consider both physiological and psychological well-being [16,17].

Emotional and psychological outcomes for ICU patients have also been studied extensively. Rattray et al. reported that anxiety, depression, and posttraumatic stress following ICU discharge were correlated with age, sex, and total hospitalization duration [18]. Similarly, Russell reported that effective communication between ICU teams and patients significantly reduced concerns about treatment, alleviated anxiety, and improved patient experiences [19]. These findings underscore the importance of addressing both physiological and psychological needs during intensive care unit (ICU) care.

In our study, age, hospitalization duration, and laboratory results aligned with expectations regarding their impact on questionnaire outcomes. Correlation analyses revealed that elderly patients exhibited lower awareness, recall, and satisfaction with care but reported more frightening experiences—trends that were also observed with longer hospitalization durations. Renal function parameters, such as creatinine and BUN, were associated with a single negative correlation, whereas albumin and nutritional support demonstrated opposite trends. These findings validated the reliability of our regression models and the inclusion of additional parameters in the evaluation of ICU experience.

Age emerged as a particularly significant factor. Although it was correlated with all four questionnaire subgroups, regression analysis revealed that frightening experience was the only subgroup in which age retained statistical significance, with elderly patients reporting more frequent frightening experiences regardless of other parameters. This suggests that older ICU patients may experience heightened stress due to cognitive impairment, reduced adaptability to the ICU environment, or prior negative healthcare experiences [20]. Implementing targeted interventions, such as psychological support or improved communication strategies, may help alleviate distress in older patients. Similarly, sex was significant, with male patients showing lower awareness than female patients during the ICU stay.

Among the laboratory parameters, magnesium was the only independent factor identified in our study. Hypomagnesemia was negatively associated with awareness, highlighting its potential role in ICU outcomes. Magnesium is the second most abundant intracellular cation and plays a crucial role in immune regulation and homeostasis [21,22]. Francesco et al. emphasized the importance of magnesium in ICU patients, noting that hypomagnesemia is associated with increased risks of infection, sepsis, weakened respiratory muscles, and bronchospasm, which can ultimately reduce survival rates [23]. However, overcorrection leading to hypermagnesemia may result in adverse effects, including paralysis, bradycardia, respiratory failure, and cardiac arrest. Beyond its statistical relevance, alterations in magnesium levels could reflect electrolyte imbalances due to disease severity or therapeutic interventions [24]. Further studies are needed to clarify the optimal magnesium correction strategies and their impact on respiratory failure requiring mechanical ventilation. In our study, magnesium was negatively associated with awareness, but its effects were limited to this subgroup, whereas nutritional support influenced all four subgroups.

The literature and the findings of our study indicate that, to improve ICU experiences, it is crucial to optimize the psychological and physiological well-being of patients requiring additional nutritional support under ICU conditions. Even if swallowing function is unaffected, it is unrealistic to expect that patients with pain and anxiety symptoms can meet their caloric and protein needs solely through oral nutritional support. Furthermore, we believe that in elderly patients, greater family support may help prevent frightening experiences in the ICU setting.

## 5. Conclusions

These findings reinforce the assumption that nutritional support is a critical factor in the ICU experience questionnaire, regardless of a patient’s nutritional status prior to admission. As shown in Table 2, a limitation of this study is the similarity in laboratory findings between admission and discharge. This limitation highlights the potential influence of nutritional support within a relatively homogenous patient population and suggests that the findings may not be generalizable to patients with better or worse nutritional status at admission. Future research should incorporate more diverse patient groups and explore whether different ICU nutritional protocols yield varying patient experiences.

### Study Limitations and Future Research

While our study provides valuable insights into the impact of nutritional support on ICU experiences, certain limitations should be acknowledged. First, our sample size was limited to a single-center cohort, which may restrict the generalizability of our findings. Second, despite our statistical analyses, unmeasured confounding factors such as sedation levels, comorbid conditions, and prior psychological health may have influenced patient perceptions. Finally, we did not assess long-term outcomes after ICU discharge, which could provide a more comprehensive understanding of post-ICU recovery.

## Figures and Tables

**Table 1:** Demographic information and hospitalization parameters.

Demographic Data, Hospitalization Status, Nutritional Evaluation, and Comorbidities		No. of Patients (*n* = 171) (%)
Gender	Male	113 (66.1)
	Female	58 (33.9)
Age	(Mean, SD)	68.25 (±11.28)
Education	No formal education	32 (18.7)
	Primary–middle school	125 (73.1)
	High school	12 (7.0)
	College–university	2 (1.2)
Comorbidities	Hypertension	75 (43.9)
	Diabetes mellitus	43 (25.1)
	Congestive heart failure	30 (17.5)
	Coronary arterial disease	15 (8.8)
	Kyphosis	3 (1.8)
	Idiopathic pulmonary fibrosis	1 (0.6)
Hospitalization and nutritional parameters	Hospitalization duration (days) (mean, SD)	10.3 (±6.2)
	NIMV requirement	108 (63.2)
	BMI upon ICU admission (mean, SD)	24.28 (±3.78)
	Enteral support	26 (15.2)
	Parenteral support	24 (14)
ICU admission origin	Ward	17 (9.9)
	Emergency service	77 (45)
	Other ICU	77 (45)

SD: standard deviation, BMI: body mass index, NIMV: noninvasive mechanical ventilation, ICU: intensive care unit. Other ICU defined to include patient admission from other ICU units within the same hospital.

**Table 2:** Comparison of laboratory parameters between intensive care unit admission and discharge patients.

Parameters	Testing Time	Mean	Standard Deviation	Paired Samples ***t***-Test		
				t	dF	***p***
Hemoglobin (g/dL)	Pre	11.9673	2.56991	2.362	170	**0.019**
	Post	11.6205	2.12611			
Creatinine (mg/dL)	Pre	1.0025	0.55667	2.256	170	**0.025**
	Post	0.9244	0.36253			
BUN (mg/dL)	Pre	50.0819	28.91192	1.069	170	0.287
	Post	48.1228	27.08510			
Sodium (mEq/L)	Pre	138.9471	4.66798	1.581	169	0.116
	Post	138.3824	3.49484			
Chloride (mEq/L)	Pre	97.0000	5.43843	1.052	170	0.294
	Post	96.3333	8.25405			
Potassium (mEq/L)	Pre	4.1718	0.58828	−0.587	170	0.558
	Post	4.2021	0.51713			
Magnesium (mEq/L)	Pre	2.0023	0.56287	1.422	170	0.157
	Post	1.9415	0.25153			
Albumin (g/L)	Pre	30.9789	6.24284	0.568	170	0.571
	Post	30.6936	7.40593			
Total protein (g/L)	Pre	55.7567	11.02030	0.038	170	0.970
	Post	55.7320	8.64708			

Testing time refers to the time of blood sampling, for which the initial result is taken at the time of admission, and the second result is the last blood sampling performed before intensive care unit discharge.

**Table 3:** Intensive care experience questionnaire parameters and subgroup results.

Questionnaire Components	Mean (SD)	Median	Mode
**Awareness of surroundings**			
I felt safe.	4.13 (±0.82)	4	4
I knew what was happening to me.	3.53 (±1.28)	4	5
I was aware of someone near to me.	4.43 (±0.66)	5	5
I was able to let people know what I wanted.	3.98 (±1.15)	4	5
I felt the absence of my relatives.	3.67 (±1.18)	4	4
**Recall of experiences**			
I never knew whether it was day or night.	3.50 (±1.44)	4	5
I seemed to sleep too much.	3.13 (±1.31)	3	2
Most of my memories are blurred.	3.80 (±1.02)	4	4
I felt safer in the morning.	3.29 (±1.31)	4	4
**Frightening experiences**			
I saw strange things.	3.12 (±1.09)	3	4
I felt helpless.	2.92 (±1.26)	3	4
I seemed to be in pain.	2.75 (±1.03)	3	3
I felt scared.	2.66 (±1.24)	3	4
I seemed to have bad dreams.	2.55 (±1.20)	3	2
I thought I would die.	3.25 (±1.24)	4	4
**Satisfaction with care**			
It was always too noisy.	3.23 (±1.21)	3	2
My care was as good as it could have been.	4.41 (±0.75)	5	5
I was constantly disturbed.	3.82 (±1.06)	4	4
I felt uncomfortable being dependent on meeting my needs.	2.44 (±1.29)	2	2
**Subgroup scores**	**Mean (SD)**	**Median**	**Min–max**
Awareness of surroundings	19.73 (±2.96)	20	12–25
Recall of experiences	13.73 (±2.52)	14	6–19
Frightening experiences	17.62 (±5.43)	18	6–26
Satisfaction with care	13.90 (±2.68)	14	7–20
**Total Score**	64.61 (±4.44)	64	53–78

SD: standard deviation, Min–max: minimum and maximum reported values.

**Table 4:** (**a**) Correlations between intensive care unit evaluation subgroups, total scores, and other parameters. (**b**) Correlations between intensive care unit evaluation subgroups, total scores, and other parameters.

(**a**)						
	**Pearson Correlation and ***p*** Value**	**Awareness of Surroundings**	**Recall of Experiences**	**Frightening Experiences**	**Satisfaction with Care**	**Total Score**
**Gender**	Correlation	0.136	0.019	0.034	−0.093	0.087
	*p* value	0.076	0.801	0.656	0.225	0.256
**Age**	Correlation	−0.294	−0.228	0.308	−0.193	−0.067
	*p* value	**0.001**	**0.003**	**0.001**	**0.012**	0.384
**Hospitalization days**	Correlation	−0.153	0.024	0.220	−0.225	0.044
	*p* value	**0.046**	0.760	**0.004**	**0.003**	0.567
**NIMV history**	Correlation	−0.065	−0.166	0.041	−0.010	−0.094
	*p* value	0.396	**0.030**	0.597	0.894	0.222
**Admission BMI**	Correlation	0.085	0.094	−0.112	0.060	0.009
	*p* value	0.267	0.222	0.144	0.436	0.904
**Hypertension**	Correlation	−0.111	−0.011	0.128	−0.099	0.016
	*p* value	0.149	0.885	0.096	0.197	0.837
**Diabetes mellitus**	Correlation	−0.038	0.074	0.107	−0.165	0.047
	*p* value	0.618	0.335	0.165	**0.031**	0.538
**Congestive heart failure**	Correlation	−0.124	−0.078	0.185	−0.161	0.002
	*p* value	0.105	0.310	**0.015**	**0.036**	0.979
**Coronary Heart disease**	Correlation	0.091	0.001	−0.053	0.058	0.032
	*p* value	0.236	0.990	0.492	0.453	0.681
(**b**)						
	**Pearson Correlation and ***p*** Value**	**Awareness of Surroundings**	**Recall of Experiences**	**Frightening Experiences**	**Satisfaction with Care**	**Total Score**
**Hemoglobin**	Correlation	0.070	−0.053	−0.159	0.135	−0.096
	*p* value	0.362	0.488	**0.038**	0.077	0.210
**Creatinine**	Correlation	−0.122	−0.211	0.122	−0.053	−0.084
	*p* value	0.111	**0.006**	0.112	0.490	0.274
**BUN**	Correlation	−0.183	−0.213	0.187	−0.074	−0.059
	*p* value	**0.016**	**0.005**	**0.014**	0.335	0.441
**Sodium**	Correlation	0.033	−0.001	0.003	0.000	0.025
	*p* value	0.666	0.992	0.966	0.996	0.742
**Chloride**	Correlation	−0.098	−0.007	0.208	−0.151	0.093
	*p* value	0.200	0.929	**0.006**	**0.049**	0.224
**Potassium**	Correlation	−0.014	0.037	−0.090	0.044	−0.072
	*p* value	0.858	0.627	0.239	0.565	0.351
**Magnesium**	Correlation	−0.163	−0.058	0.088	−0.066	−0.074
	*p* value	**0.033**	0.451	0.251	0.389	0.337
**Albumin**	Correlation	0.182	0.130	−0.251	0.197	0.008
	*p* value	**0.017**	0.089	**0.001**	**0.010**	0.922
**Total protein**	Correlation	−0.013	−0.025	−0.041	−0.045	−0.099
	*p* value	0.871	0.749	0.597	0.561	0.197
**Nutritional support**	Correlation	−0.440	−0.301	0.467	−0.265	−0.053
	*p* value	**0.001**	**0.001**	**0.001**	**0.001**	0.492

NIMV: noninvasive mechanical ventilation, BMI: body mass index, diabetes mellitus (DM) diagnosis includes formerly diagnosed type 1 and type 2 DM. BUN: blood urea nitrogen.

**Table 5:** (**a**) Regression analysis between awareness of surroundings, recall of experiences and other parameters. (**b**) Regression analysis between awareness of surroundings, recall of experiences, and other parameters. (**c**) Regression analysis between awareness of surroundings, recall of experiences, and other parameters. (**d**) Regression analysis between awareness of surroundings, recall of experiences, and other parameters.

(**a**)				
**Awareness of Surroundings as the Dependent Variable**	***B***	***Standard Error***	***t***	***p Value***
***Constant***	14.306	7.682	1.862	0.065
**Gender**	0.991	0.474	2.092	**0.038**
**Age**	−0.041	0.021	−1.928	0.056
**Hospitalization days**	−0.020	0.035	−0.560	0.576
**NIMV history**	−0.082	0.448	−0.183	0.855
**Admission BMI**	0.033	0.056	0.590	0.556
**Hypertension**	−0.396	0.450	−0.880	0.380
**Diabetes mellitus**	0.672	0.509	1.321	0.189
**Congestive heart failure**	−0.455	0.597	−0.763	0.447
**Coronary heart disease**	0.406	0.756	0.538	0.591
(**b**)				
**Awareness of Surroundings as the Dependent Variable**	***B***	***Standard Error***	***t***	***p Value***
**Hemoglobin**	0.062	0.092	0.679	0.498
**Creatinine**	0.223	0.440	0.506	0.613
**BUN**	−0.001	0.009	−0.093	0.926
**Sodium**	0.075	0.052	1.443	0.151
**Chloride**	−0.006	0.043	−0.147	0.884
**Potassium**	0.133	0.424	0.314	0.754
**Magnesium**	−1.231	0.382	−3.221	**0.002**
**Albumin**	0.045	0.039	1.156	0.250
**Total protein**	−0.012	0.022	−0.561	0.576
**Nutritional support**	−2.629	0.559	−4.706	**0.001**
**Recall of experiences as the dependent variable**				
(**c**)				
**Awareness of Surroundings as the Dependent Variable**	***B***	***Standard Error***	***t***	***p Value***
***Constant***	5.415	6.965	0.778	0.438
**Gender**	−0.345	0.430	−0.802	0.424
**Age**	−0.023	0.019	−1.196	0.233
**Hospitalization days**	0.048	0.032	1.517	0.131
**NIMV history**	−0.634	0.406	−1.561	0.121
**Admission BMI**	0.067	0.051	1.316	0.190
**Hypertension**	0.274	0.408	0.672	0.503
**Diabetes mellitus**	0.709	0.461	1.538	0.126
**Congestive heart failure**	−0.180	0.541	−0.334	0.739
**Coronary heart disease**	0.349	0.685	0.509	0.611
(**d**)				
**Awareness of Surroundings as the Dependent Variable**	***B***	***Standard Error***	***t***	***p Value***
**Hemoglobin**	−0.098	0.083	−1.181	0.239
**Creatinine**	−0.448	0.399	−1.122	0.264
**BUN**	−0.005	0.008	−0.601	0.549
**Sodium**	0.047	0.047	0.989	0.324
**Chloride**	0.046	0.039	1.172	0.243
**Potassium**	0.329	0.384	0.858	0.392
**Magnesium**	−0.350	0.346	−1.009	0.314
**Albumin**	0.047	0.035	1.335	0.184
**Total protein**	−0.019	0.020	−0.929	0.354
**Nutritional support**	−1.951	0.507	−3.851	**0.001**

NIMV: noninvasive mechanical ventilation, BMI: body mass index, BUN: blood urea nitrogen.

**Table 6:** Regression analysis between frightening experiences, satisfaction with care, and other parameters.

Frightening Experiences as the Dependent Variable	***B***	***Standard Error***	***t***	***p Value***
***Constant***	17.423	13.842	1.259	0.210
**Gender**	0.126	0.854	0.148	0.882
**Age**	0.077	0.038	2.006	**0.047**
**Hospitalization days**	0.062	0.063	0.991	0.323
**NIMV history**	0.384	0.808	0.476	0.635
**Admission BMI**	−0.150	0.102	−1.478	0.142
**Hypertension**	0.031	0.811	0.038	0.970
**Diabetes mellitus**	−0.234	0.917	−0.256	0.798
**Congestive heart failure**	1.482	1.076	1.378	0.170
**Coronary heart disease**	−0.052	1.362	−0.038	0.970
**Hemoglobin**	−0.152	0.165	−0.922	0.358
**Creatinine**	−0.133	0.793	−0.167	0.867
**BUN**	−0.012	0.016	−0.751	0.454
**Sodium**	−0.133	0.094	−1.413	0.160
**Chloride**	0.138	0.078	1.766	0.079
**Potassium**	−1.063	0.764	−1.392	0.166
**Magnesium**	1.730	0.688	2.512	0.013
**Albumin**	−0.088	0.070	−1.253	0.212
**Total protein**	0.013	0.040	0.337	0.737
**Nutritional support**	4.693	1.007	4.662	**0.001**
**Satisfaction with care as the dependent variable**				
***Constant***	19.276	7.637	2.524	0.013
**Gender**	−0.338	0.471	−0.717	0.475
**Age**	−0.018	0.021	−0.844	0.400
**Hospitalization days**	−0.053	0.035	−1.536	0.127
**NIMV history**	−0.084	0.446	−0.188	0.851
**Admission BMI**	0.046	0.056	0.827	0.410
**Hypertension**	0.089	0.447	0.200	0.842
**Diabetes mellitus**	−0.414	0.506	−0.819	0.414
**Congestive heart failure**	−0.678	0.593	−1.143	0.255
**Coronary heart disease**	0.164	0.751	0.218	0.828
**Hemoglobin**	0.044	0.091	0.481	0.631
**Creatinine**	−0.001	0.437	−0.003	0.998
**BUN**	0.009	0.009	1.041	0.299
**Sodium**	0.017	0.052	0.321	0.748
**Chloride**	−0.059	0.043	−1.377	0.170
**Potassium**	0.317	0.421	0.753	0.453
**Magnesium**	−0.618	0.380	−1.627	0.106
**Albumin**	0.066	0.039	1.714	0.089
**Total protein**	−0.025	0.022	−1.140	0.256
**Nutritional support**	−1.137	0.555	−2.047	**0.042**

NIMV: noninvasive mechanical ventilation, BMI: body mass index, BUN: blood urea nitrogen.

## Data Availability

Our data cannot be shared openly to protect study participant privacy. If requested, the data will be shared to review statistical evaluation for meta-analysis and other scientific reasons.
